# Correction: The zinc finger proteins ZNF644 and WIZ regulate the G9a/GLP complex for gene repression

**DOI:** 10.7554/eLife.08168

**Published:** 2015-04-22

**Authors:** Chunjing Bian, Qiang Chen, Xiaochun Yu

Bian C, Chen Q, Yu X. 2015. The zinc finger proteins ZNF644 and WIZ regulate the G9a/GLP complex for gene repression. *eLife* **4**:e05606. doi: 10.7554/eLife.05606

Published 19 March 2015

There was an error in the WIZ binding sequence used for the EMSA experiments. The incorrect sequence was 5′-GAGTCTCACTCACGCGC***TTCGATGATTCC***CAGATACTAGTACGGTCAG-3′ (WIZ ‘WT DNA target’).

The correct sequence is 5′-GAGTCTCACTCACGCGC***CATTCCATTCCATT***CAGATACTAGTACGGTCAG-3′ (WIZ ‘WT DNA target’).

The article has been corrected accordingly.

